# Supplementary figures and images for: Systems genetics in the rat HXB/BXH family identifies Tti2 as a pleiotropic quantitative trait gene for adult hippocampal neurogenesis and serum glucose
Source: PLoS Genet. 2022 Apr 4;18(4):e1009638. doi: 10.1371/journal.pgen.1009638 (PMC9060359; doi:10.1371/journal.pgen.1009638)

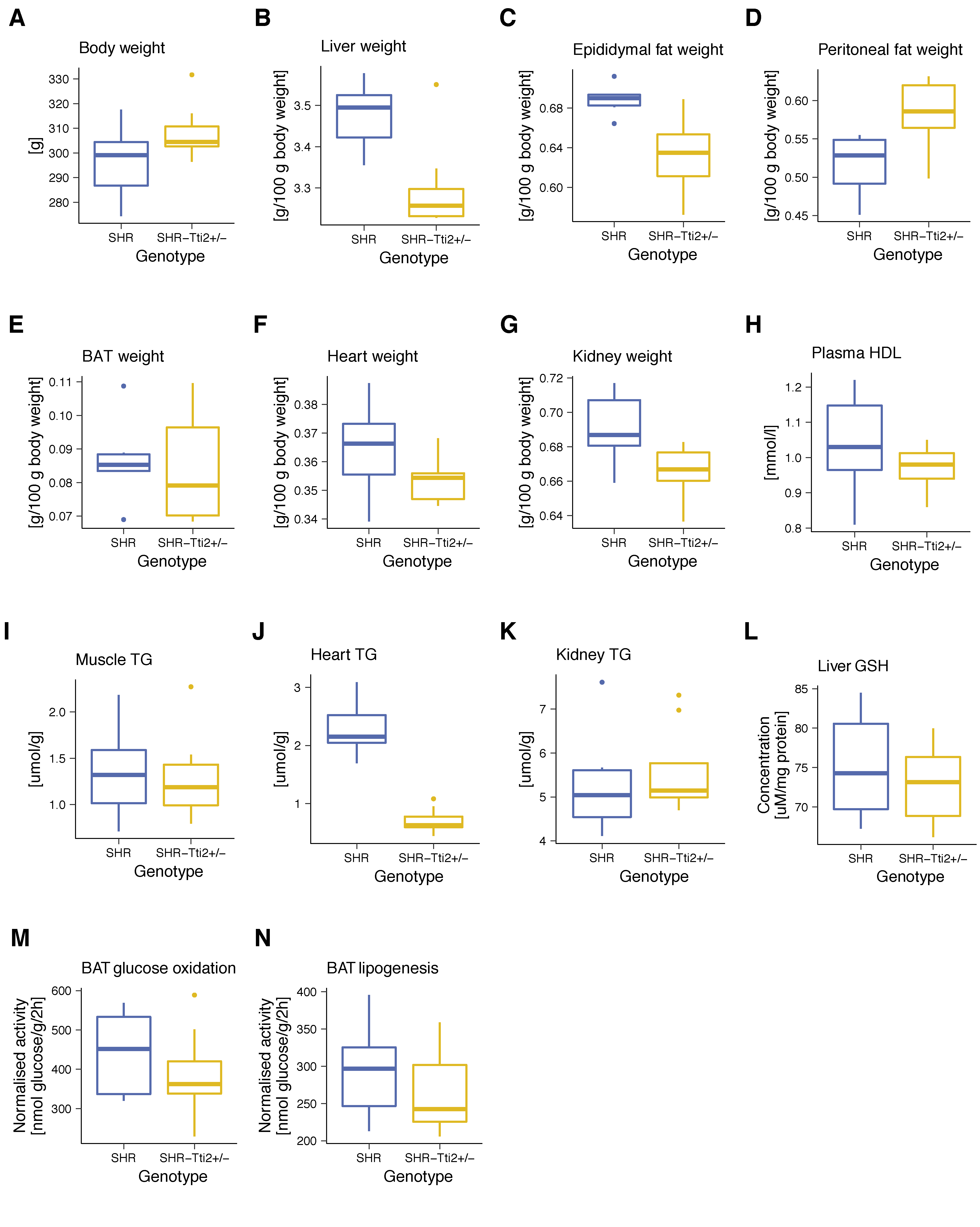

Supplement: S1 Fig — Details of statistical analysis are in Table 2. Abbreviations: BAT, brown adipose tissue; GSH, glutathione; HDL, high-density lipoprotein; TG, triglycerides. (TIF) [file pgen.1009638.s001.tif]

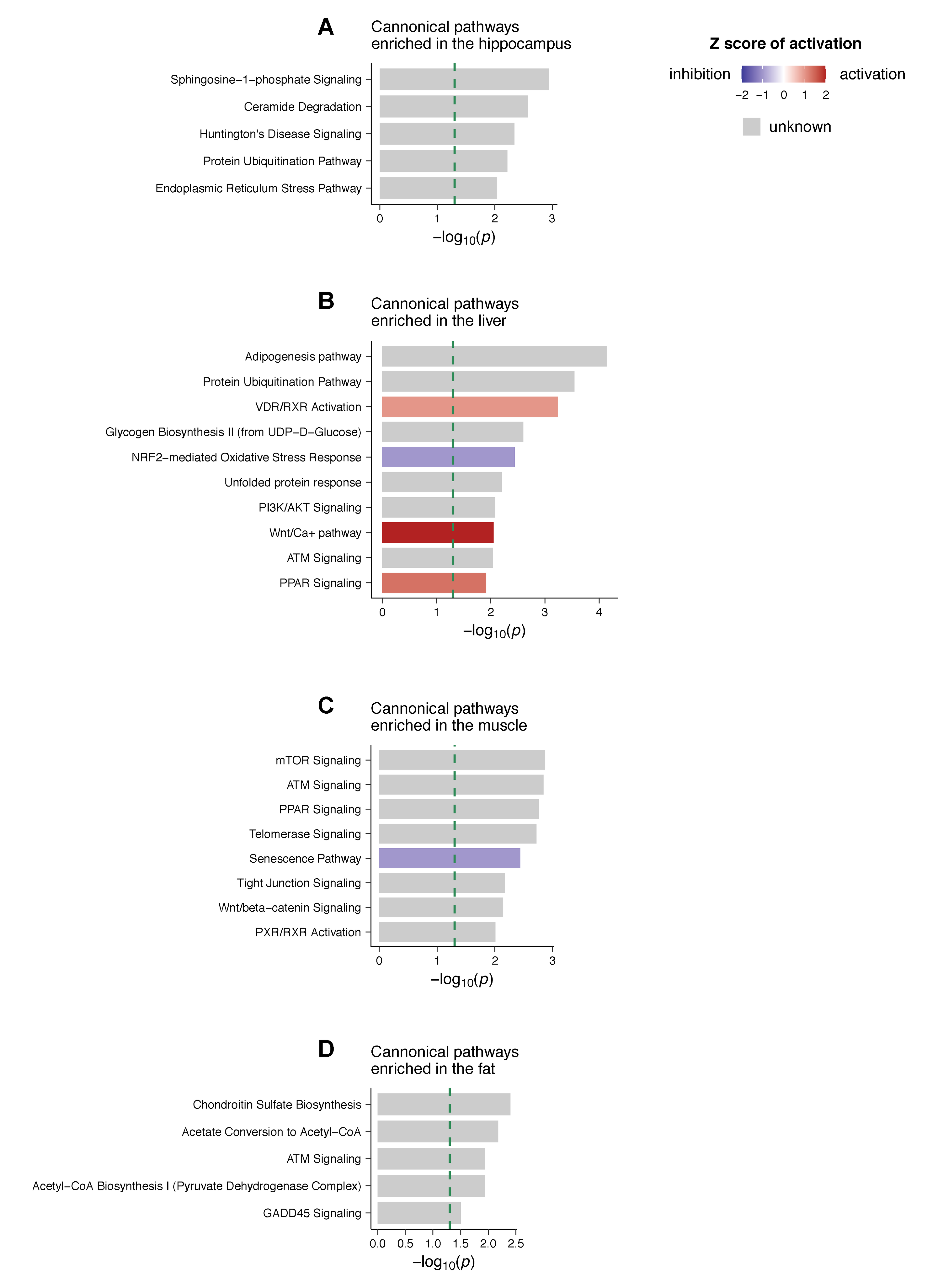

Supplement: S2 Fig — Analysis was performed with Ingenuity pathway analysis (IPA), which, in addition to calculating enrichment, predicts activation (positive Z-score, red) or inhibition (negative Z-score, blue) of molecular pathways from the direction and magnitude of expression changes using curated database. Grey bars depict enriched pathways for which activation status could not be predicted. Vertical green dashed line indicates p value threshold of 0.05. (TIF) [file pgen.1009638.s002.tif]

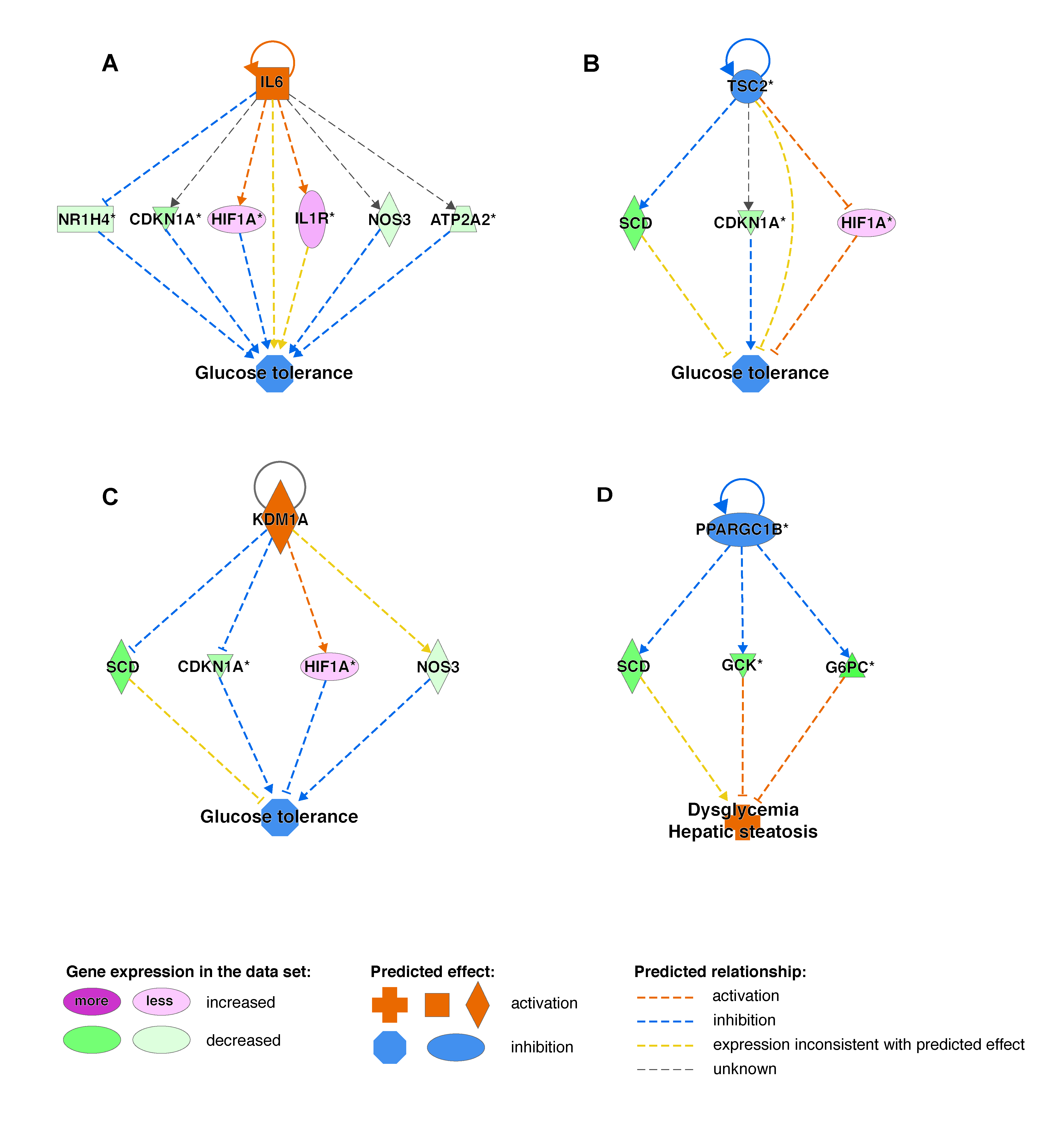

Supplement: S3 Fig — Differentially expressed genes in livers of SHR-Tti2+/- rats and wild type SHR littermates were analysed using IPA. Up- (magenta) and downregulated genes (green; middle tier) connect the potential upstream regulators (upper tier) to downstream outcomes (bottom tier). Edges represent relationships derived from curated databases. (TIF) [file pgen.1009638.s003.tif]

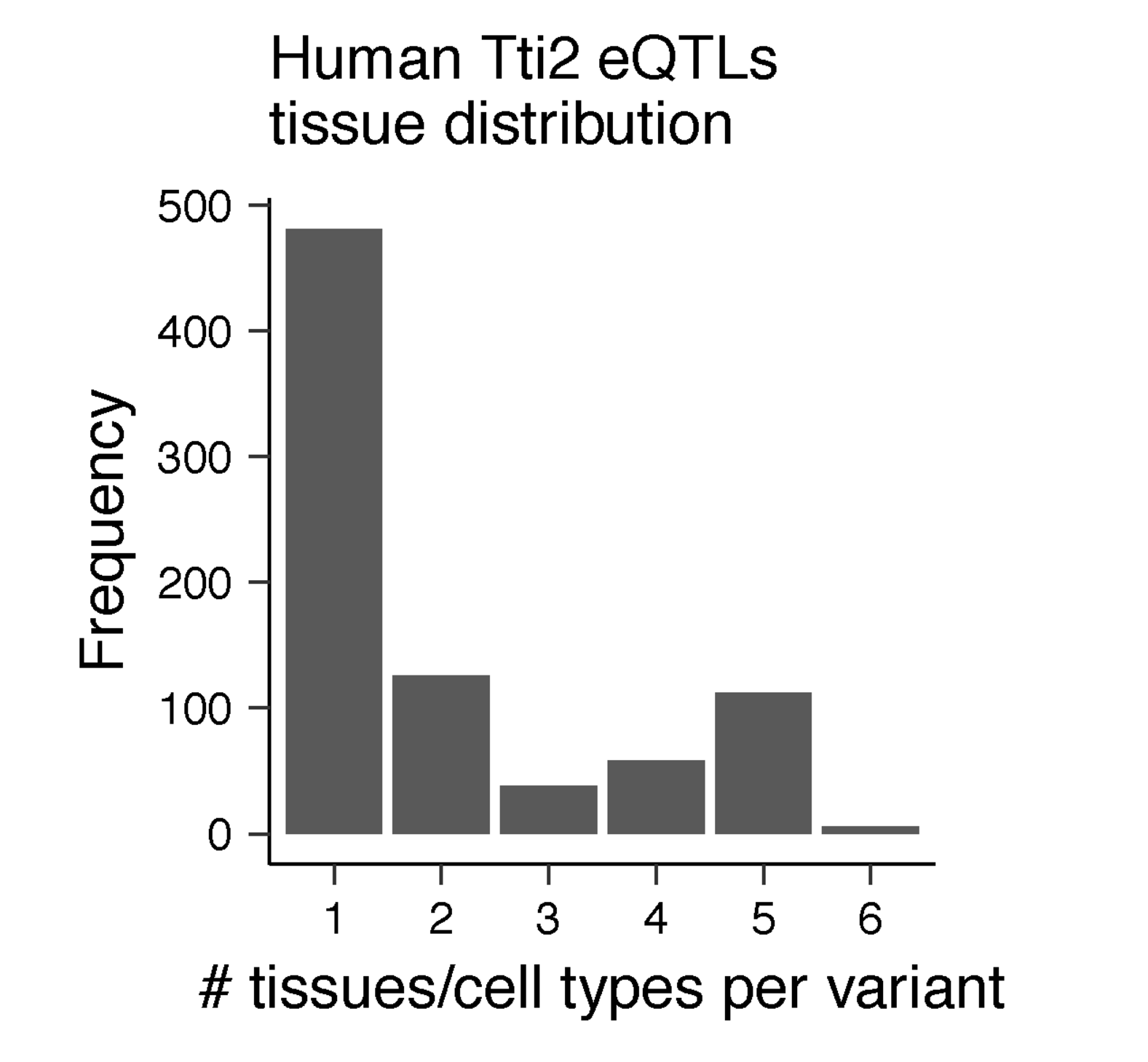

Supplement: S4 Fig — 821 genomic variants underlying 2177 eQTL (p < 1e-4) extracted from eQTL EBI catalogue (https://www.ebi.ac.uk/eqtl/) were clustered according to the number of distinct tissues or cell types in which eQTL were detected. Multiple eQTL from different data sets derived from the same cell or tissue type were scored as a single-tissue eQTL. (TIF) [file pgen.1009638.s004.tif]

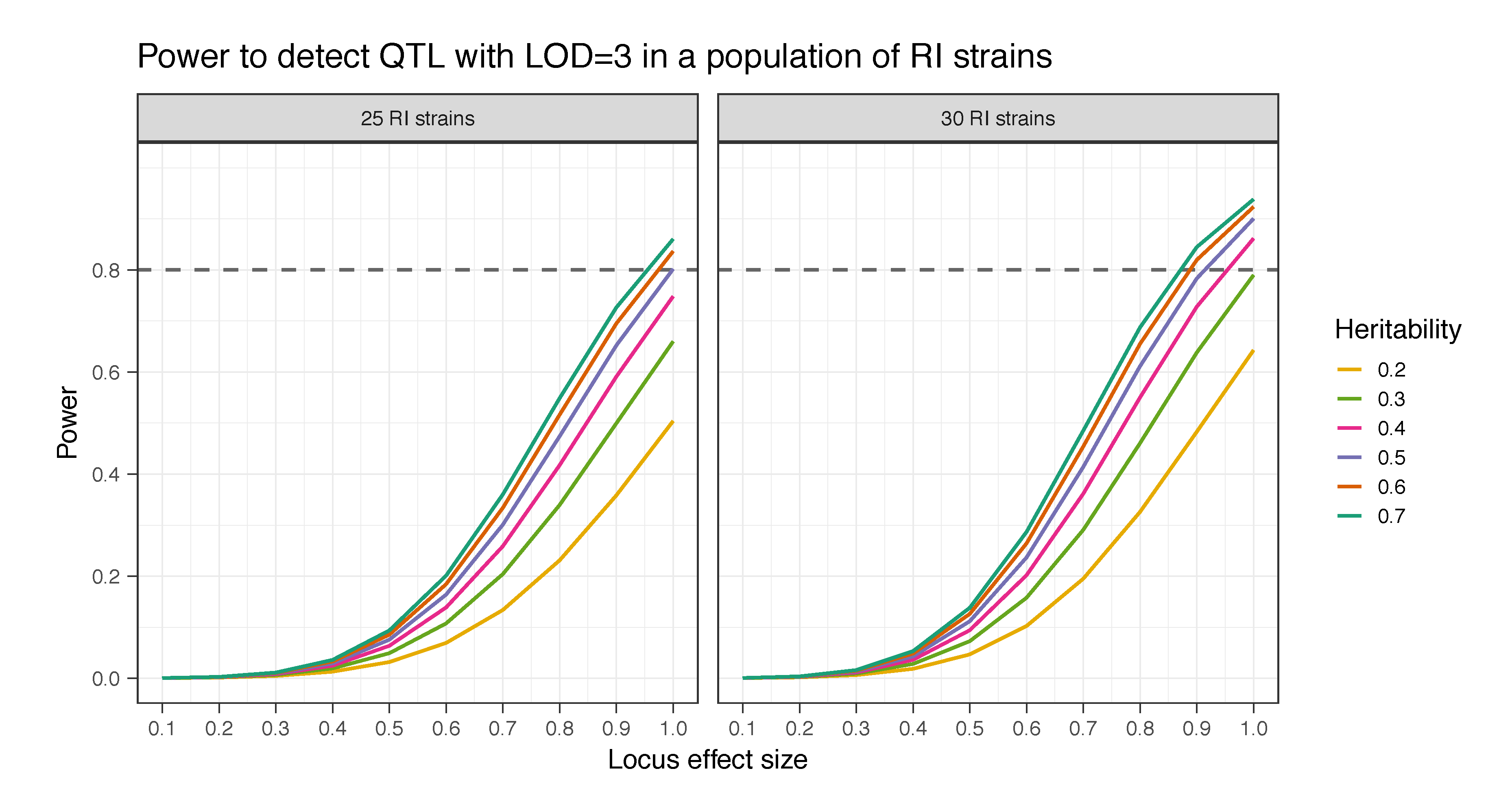

Supplement: S5 Fig — Graphs depict theoretical calculations of power to detect a QTL with a minimum LOD value of 3 with various genetic effect sizes of a locus and heritability ranging from 0.2 to 0.7. Effect size is a difference in mean trait value between homozygous animals with different parental allele at this locus expressed as a proportion of the total genetic variance. Effect size of 1 implies a Mendelian trait. The calculations were performed for 25 and 30 lines with 5 biological replicates each, because several of the published traits used in our study, including serum glucose levels, were not measured in the entire family. (TIF) [file pgen.1009638.s005.tif]
