# Supplementary material for: Systems genetics in the rat HXB/BXH family identifies Tti2 as a pleiotropic quantitative trait gene for adult hippocampal neurogenesis and serum glucose
Source: PLoS Genet. 2022 Apr 4;18(4):e1009638. doi: 10.1371/journal.pgen.1009638 (PMC9060359; doi:10.1371/journal.pgen.1009638)
Supplement: S1 Table — (DOCX) [file pgen.1009638.s011.docx]

| RGD ID | Ensembl Gene ID | Symbol | Name | Type | Start | Stop |
| --- | --- | --- | --- | --- | --- | --- |
| 1305088 | ENSRNOG00000015426 | Purg | purine-rich element binding protein G | protein-coding | 62094495 | 62148088 |
| 1564788 | ENSRNOG00000015440 | Wrn | WRN RecQ like helicase | protein-coding | 62148560 | 62283748 |
| 1563097 |  | RGD1563097 | similar to ribosomal protein S15a | protein-coding | 62293104 | 62293541 |
| 621341 | ENSRNOG00000010392 | Nrg1 | neuregulin 1 | protein-coding | 62632432 | 63718738 |
| 1359164 | ENSRNOG00000050857 | Fut10 | fucosyltransferase 10 | protein-coding | 64290270 | 64373665 |
| 1311297 | ENSRNOG00000010783 | Mak16 | MAK16 homolog | protein-coding | 64386704 | 64395257 |
| 1310414 | ENSRNOG00000023494 | Tti2 | TELO2 interacting protein 2 | protein-coding | 64394792 | 64402657 |
| 1561238 | ENSRNOG00000023473 | Rnf122 | ring finger protein 122 | protein-coding | 64424229 | 64429199 |
| 1310090 | ENSRNOG00000011518 | Dusp26 | dual specificity phosphatase 26 | protein-coding | 64455731 | 64463284 |
| 1562800 | ENSRNOG00000045737 | LOC302192 | similar to RIKEN cDNA 1700001E04 | protein-coding | 66002994 | 66024065 |
| 7659612 |  | LOC102554887 | uncharacterized LOC102554887 | non-coding RNA | 62112832 | 62117250 |
| 7578262 |  | LOC102555473 | uncharacterized LOC102555473 | non-coding RNA | 63746258 | 63746863 |
| 7746793 |  | LOC102555712 | uncharacterized LOC102555712 | non-coding RNA | 64447281 | 64451810 |
| 7543674 |  | LOC102556287 | uncharacterized LOC102556287 | non-coding RNA | 64827697 | 64901092 |
