# Supplementary material for: Systems genetics in the rat HXB/BXH family identifies Tti2 as a pleiotropic quantitative trait gene for adult hippocampal neurogenesis and serum glucose
Source: PLoS Genet. 2022 Apr 4;18(4):e1009638. doi: 10.1371/journal.pgen.1009638 (PMC9060359; doi:10.1371/journal.pgen.1009638)
Supplement: S2 Table — Expression of genes whose cis-eQTL were located within the joint neurogenesis-serum glucose QTL interval on Chromosome 16 (Chr 16: 62.1–66.3 Mb), or within neurogenesis-specific QTL interval (Chr 16: 56.8–74.3), were used for conditional mapping for these phenotypes. Although Saraf gene is located outside the joint phenotype QTL interval, it has a cis-eQTL within the QTL region. Conditional LOD scores and the respective difference in phenotype LOD scores for each gene are noted. Pearson’s correlation coefficient values denote correlation between a phenotype and gene expression in listed tissues. (DOCX) [file pgen.1009638.s012.docx]

| Phenotype | Gene | cis-eQTL LOD | eQTL position | Tissue | Phenotype LOD | Conditional LOD | LOD change | Pearson’s *r* |
| --- | --- | --- | --- | --- | --- | --- | --- | --- |
| BrdU | *Tti2* | 4.06 | 64 | hippocampus | 3.12 | 0.78 | -2.34 | -0.65 |
|  | *Tti2* | 14.42 | 64.1 | Tti2 eigengene |  | 1.09 | -2.03 | -0.61 |
|  | *Mak16* | 6.11 | 64.2 | muscle |  | 3.16 | 0.22 | 0.02 |
|  | *Gsr* | 3.03 | 63.6 | hippocampus |  | 2.47 | -0.65 | -0.38 |
|  | *Adam32* | 3.03 | 72.4 | hippocampus |  | 2.8 | -0.32 | 0.23 |
|  | *Htra4* | 5.22 | 72.4 | hippocampus |  | 2.81 | -0.31 | 0.20 |
| Serum glucose | *Tti2* | 14.42 | 64.1 | Tti2 eigengene | 5.13 | 2.37 | -2.76 | -0.72 |
|  | *Tti2* | 7.16 | 64.1 | adrenal gland |  | 3.79 | -1.34 | -0.64 |
|  | *Tti2* | 10.8 | 64.2 | aorta |  | 3.43 | -1.7 | -0.63 |
|  | *Tti2* | 10.66 | 64.2 | fat |  | 4.19 | -0.94 | -0.55 |
|  | *Tti2* | 7.72 | 66.3 | kidney |  | 3.03 | -2.1 | -0.77 |
|  | *Tti2* | 12.5 | 64.2 | liver |  | 3.62 | -1.51 | -0.6 |
|  | *Tti2* | 18.7 | 64.2 | muscle |  | 2.06 | -3.07 | -0.69 |
|  | *Tti2* | 9.53 | 64.2 | ventricle |  | 1.64 | -3.49 | -0.77 |
|  | *Mak16* | 6.11 | 64.2 | muscle |  | 2.82 | -2.31 | -0.59 |
|  | *Saraf* | 4.4 | 62.3 | kidney |  | 6.28 | 1.15 | -0.07 |
