# Supplementary material for: Systems genetics in the rat HXB/BXH family identifies Tti2 as a pleiotropic quantitative trait gene for adult hippocampal neurogenesis and serum glucose
Source: PLoS Genet. 2022 Apr 4;18(4):e1009638. doi: 10.1371/journal.pgen.1009638 (PMC9060359; doi:10.1371/journal.pgen.1009638)
Supplement: S3 Table — Expression values were normalised to the mean expression of Tti2 in BN. Note that lower cycle of threshold (ΔΔCT) values indicate higher relative expression of a gene. Data is shown as means ± standard error of the mean; t, Student’s t-test statistic; df, degrees of freedom. (DOCX) [file pgen.1009638.s013.docx]

| Tissue | ∆∆CT (SHR – BN) | *t* | df | *p*-value |
| --- | --- | --- | --- | --- |
| Adrenal gland | -0.39 ± 0.13 | 1.84 | 9 | 0.099 |
| Perirenal fat | 1.15 ± 0.13 | -4.83 | 8 | 0.0013 |
| Hippocampus | -0.88 ± 0.1 | 3.5 | 8 | 0.0081 |
| Kidney | -1.14 ± 0.12 | 5.31 | 8 | 0.00072 |
| Liver | -0.85 ± 0.08 | 2.77 | 9 | 0.022 |
| Soleus muscle | -1.49 ± 0.15 | 7.34 | 8 | 8.10E-05 |
| Pancreas | -1.15 ± 0.11 | 2.49 | 8 | 0.037 |
