# Supplementary material for: Systems genetics in the rat HXB/BXH family identifies Tti2 as a pleiotropic quantitative trait gene for adult hippocampal neurogenesis and serum glucose
Source: PLoS Genet. 2022 Apr 4;18(4):e1009638. doi: 10.1371/journal.pgen.1009638 (PMC9060359; doi:10.1371/journal.pgen.1009638)
Supplement: S4 Table — Genomic sequence of SHR rats between positions 62.1 and 66.3 Mb on chromosome 16, which cover neurogenesis-glucose QTL, was scanned for non-synonymous amino-acid substitutions compared to reference genome using Variant Visualiser in Rat Genome Database. Within this interval, missense mutations were present only in the Tti2 gene. Conservation score ranges from 1 (highly conserved) to 0 (not conserved). SIFT score ranges from 0 (damaging) to 1 (non-damaging). Positions are according to Rnor_5.0 genome assembly. (DOCX) [file pgen.1009638.s014.docx]

| Position | Conservation Score | Reference | SHR | Reference Amino Acid | Variant Amino Acid | Amino Acid Coordinate | Polyphen Prediction | SIFT Prediction |
| --- | --- | --- | --- | --- | --- | --- | --- | --- |
| 64399852 | 0.909 | T | G | E | D | 247 | benign | 0.15 |
| 64401758 | 0.277 | C | T | A | T | 205 | benign | 0.71 |
| 64401779 | 0.567 | T | C | K | E | 198 | benign | 1 |
| 64402213 | 0.005 | C | T | R | K | 53 | benign | 0.9 |
| 64402331 | 0.001 | A | G | C | R | 14 | benign | 0.2 |
| 64402360 | 0 | C | T | G | D | 4 | benign | 1 |
