# Supplementary material for: Systems genetics in the rat HXB/BXH family identifies Tti2 as a pleiotropic quantitative trait gene for adult hippocampal neurogenesis and serum glucose
Source: PLoS Genet. 2022 Apr 4;18(4):e1009638. doi: 10.1371/journal.pgen.1009638 (PMC9060359; doi:10.1371/journal.pgen.1009638)
Supplement: S5 Table — (DOCX) [file pgen.1009638.s015.docx]

| Tissue | beta | se | *p* value | *q* value |
| --- | --- | --- | --- | --- |
| Hippocampus | -0.54 | 0.07 | 1.40E-15 | 2.8E-11 |
| Liver | -0.53 | 0.08 | 7.7E-11 | 1.8E-07 |
| Fat | -0.46 | 0.07 | 1.7E-11 | 3.5E-07 |
| Muscle | -0.63 | 0.06 | 3.70E-22 | 6.7E-18 |
