# Supplementary material for: Systems genetics in the rat HXB/BXH family identifies Tti2 as a pleiotropic quantitative trait gene for adult hippocampal neurogenesis and serum glucose
Source: PLoS Genet. 2022 Apr 4;18(4):e1009638. doi: 10.1371/journal.pgen.1009638 (PMC9060359; doi:10.1371/journal.pgen.1009638)
Supplement: Attachment — Submitted filename: 220201_PLoS Genetics_ResponseLetter.docx [file pgen.1009638.s016.docx]

RESPONSE TO REVIEWS

We would like to thank the reviewers for the insightful and constructive comments. Our response to the questions and concerns, which are highlighted in a blue font is given point by point below in a black font.

Reviewer #1

STRENGTHS

• Efficient use of resources with this GRP (eQTLs, correlated traits in database) to unbiasedly identify correlated traits (neurogenesis, metabolic) and validate these directly in the gene mutants they generated.

• N=5-9 rats per strain for 30 or so inbred strains over 3 independent litters. 3 IP injections of BRDU (50 mg/kg), sac’ed 28 days later, histological analysis.

• Het-het breeding and Littermate controls for the KO study. 50% knockdown more closely mimics the likely human condition. Why, however, were homozygous mutants not tested? Were they not viable?

• Conditioning on gene expression (Rather than markers) is a more direct, causal assessment of the eQTL and gene in trait variation.

• Extensive analysis of Tti2 mutants at multiple levels of intermediate phenotypes that were informed by the correlations identified in the GRP – gene expression (legacy and new microarray; RNA-seq in mutants), glucose utilization, oxidative stress markers (enzyme activity), basal and insulin-stimulated glycogen synthesis, biochemical (glucose, triglycerides, cholesterol, glycerol, insulin)

• Dose-dependent effects of Tti2 on HC neurogenesis and glucose homeostasis

• Striking degree of co-mapping of metabolic, neurogenesis, and Tti2 expression strengthens the case for Tti2.

• Importantly, proliferation did not map to chr. 16, indicating some degree of specificity for neurogenesis.

• Neat that conditioning on glucose levels decreased the LOD score below significance. Strengthens the argument that a shared genetic factor in chr. 16 QTL mediates both phenotypes. Although not slam-dunk evidence (even if they’re separate QTLs, there will be a high degree of local LD), it is complementary evidence. Are there any other transcripts with cis-eQTLs within the chr.16 region? Have you tried conditioning on those?

MAJOR COMMENTS
• The word pleiotropy is used to describe the findings but is it possible that the metabolic phenotype leads to changes in neurogenesis; in this case it would be pleiotropy or would it be more of a linear causal chain of events. Do the authors have any insight as to whether there is independent, concomitant regulation of both traits (pleiotropy) or whether is a causal sequence?

Neither QTL mapping nor use of general KO strategy to evaluate the candidate gene in regulation of neurogenesis and metabolism provided the tools to unambiguously distinguish direct pleiotropy (also referred to as horizontal or mosaic) from a causal cascade (known also as vertical or relational pleiotropy). The relevance of the question of course goes beyond semantics and a discussion whether vertical effect constitutes genuine or spurious pleiotropy. The understanding of mechanisms behind genetic correlations is important both for human epidemiology, and our general insights into biology of processes under study. Notwithstanding, Tti2 does constitute a genetic link between two phenotypes, irrespective of the nature of underlying pleiotropy. Without developing a series of tissue-specific conditional knock-outs, which we considered but were beyond the scope of this study, we discussed published evidence which may hint towards direct component of the effect of *Tti2* variation on neurogenesis:

- Lack of genetic correlations of neurogenesis to peripheral insulin resistance in the HXB/BXH family.
- Lack of evidence for brain insulin resistance in the hippocampal transcriptome in the SHR-Tti2+/- rats.
- Published effects of experimental manipulations of blood sugar and insulin metabolism (hyperglycemia, diabetes, caloric restriction) have effects with opposite directions on neurogenesis than observed in our study.
- Severe neurodevelopmental phenotype in children carrying compound heterozygous or homozygous loss-of-function mutations in TTI2 or other components of TTT complex in absence of striking metabolic impairments.
- Focused expression of molecular targets of TTT complex (i.e. mTOR and ATM/ATR) in the embryonic and adult neural stem cells.
- Impairments of postnatal and adult neurogenesis in ATM/ATR KO mouse models and upon mTOR inhibition.

For clarity, we have separated the discussion about the direct vs. metabolic regulation of neurogenesis by *Tti2* in a separate section. Although we mentioned in the first version of the manuscript that we cannot distinguish between direct and metabolic effect of *Tti2* on neurogenesis, we had now disclosed this limitation explicitly in the last section of the revised text:

*Line 708*

“Another limitation of our study was the use of a general rather than tissue-specific *Tti2* knockout. This choice was dictated by the complexity of both processes under study. Glucose homeostasis is achieved by interactions between many cell types in several tissues. Adult neurogenesis is not only regulated intrinsically in neural stem cells, but is also influenced by the niche in which they are harboured, and network activity of the entire hippocampus. A classical knockout approach also reflected the presence of *Tti2* cis-eQTL in all examined tissues. Although we cannot unequivocally claim that the effect of *Tti2* knockout on neurogenesis is independent of metabolism, we have discussed literature that support the hypothesis of a direct, horizontal pleiotropic effect of *Tti2* dosage on these distinct phenotypes.”

• Is Tti2 the only eQTL within the 4 Mb interval? If not, the other cis-eQTLs could be provided for all tested tissues, along with the effect of conditioning on expression of those genes on the chr.16 QTL.

*Tti2* was the only gene with multi-tissue e-QTL. Other cis-eQTL were detected for *Mak16* in the muscle data set, whose genomic location overlaps *Tti2*, and *Saraf* in the kidney transcriptome. We used both genes in the respective tissues for conditional mapping for serum glucose. Conditioning on *Mak16* also decreased LOD score for serum glucose, but not for neurogenesis, whereas *Saraf* did not have major effect on linkage value. For completeness, we have also conditioned net neurogenesis on 3 hippocampal transcripts, whose expression had a cis-eQTL located in the broader QTL interval for this phenotype. None of these genes had significant influence on the neurogenesis LOD score. We have added this data in the supplementary Table S2 and mentioned *Mak16* in the main text.

*Line 259*

“We thus carried out conditional mapping for neurogenesis and serum glucose using expression of *Tti2*, and *Mak16* as covariates (Table S2). […] Using *Mak16* expression in the muscle as a covariate also decreased the LOD score for serum glucose by 2.31 (LOD = 2.82), but not for net neurogenesis (LOD = 3.16). The genomic locus of *Mak16* partly overlaps *Tti2* gene (Table S1), so it is plausible they share some of the regulatory elements. Based on the multi-tissue cis-eQTL and correlation of *Tti2* expression to neurogenesis and serum glucose in all data sets, we concluded it is the strongest candidate gene responsible for observed variation in both phenotypes within the joint phenotype QTL.”

• The 11 protein-coding genes and 4 noncoding RNA genes within the QTL interval should be listed somewhere as well.

We now listed genes located within the joint neurogenesis-serum glucose QTL interval in new supplementary Table S1. The LOC103693976 gene, which encodes fibroin heavy chain-like protein, had been withdrawn from the Rat Genome Database, therefore we corrected the text to mention only 10 protein-coding genes.

• Interesting that neg. correlation was observed for Tti2 expression vs. neurogenesis in the GRP, yet decreased expression in the Tti2 mutants resulted in decreased neurogenesis. Could there be a dominant negative effect with one or more of the coding polymorphisms that explains this discrepancy between the GRP and the mutants?

If the effect of SHR variants within the coding sequence of *Tti2* were *dominant* negative, we would expect no difference in measured phenotypes (serum glucose and neurogenesis) when one copy of the mutant gene is removed. However values of both phenotypes decreased even further in heterozygous knock-out rats, speaking rather against negative dominant allele. We currently do not have a better explanation for the discrepancy than a speculation that some of the amino-acid substitutions in the SHR protein are partly damaging, despite that they were scored as benign by SIFT and Polyphen; and that the mRNA expression differences seen between two genotypes are independent of the protein function. There is a slim possibility that the truncated N-terminus of Tti2 is to some degree expressed and has an ectopic dominant negative role, but reduced level of mRNA in the heterozygous animals implicates nonsense-mediated mRNA decay.

The question about dominant nature of SHR variants could potentially be answered with a congenic strain by comparing heterozygous vs. homozygous animals with respect to the SHR Tti2 allele. Having choice between fine-mapping and use of congenic or targeted KO, we chose to specifically mutate *Tti2*. We explain further in response to the second reviewer our rationale for the chosen strategy to validate the candidate gene.

• If the only missense mutations within this entire 4+Mb interval are within one gene – Tti2, then I don’t think this GRP is as representative of human genetic diversity as the authors claim in the Introduction. How many polymorphisms segregate in this GRP?

Between SHR and BN autosomal genomes, there are 3.2 million single nucleotide variants, and ca. 428 thousand structural genetic variants (Simonis et al., Genome Biology 2012), which is indeed less than in human populations. We have removed this notion from the Introduction, and included the information about the number of variants in each category. The 4.2 Mb QTL interval contains 6786 variants (SNP and small insertions/deletions). However, the majority of them lie outside of the coding sequence, and out of 10 protein-coding genes located within this interval, the only amino-acid substitutions in the HXB panel were located in Tti2 gene. We have modified the Results section to reflect this information

*Line 230*

“The eigenphenotype QTL (LOD = 4.62, *p* = 0.014) spanned 4.2 Mb from genomic position 62.1 to 66.3 Mb and contained 10 protein-coding and 4 non-coding RNA genes (Table S1). The QTL covered 4983 single nucleotide variations and 1803 small insertions/deletions (≤ 10 base pairs).”

• Testing males-only is a major limitation of the study as there are known sex differences in neurogenesis. “Compatibility” with prior data and “estrus variation” are not valid scientific reasons to exclude half the population. Covariates can be included in the statistical models if necessary. If not females now for these reasons, then when?

The assessment of adult neurogenesis is a very tedious, manual process, in which sectioning of brains, staining of floating sections, mounting on glass slides and counting of stained cells required several days of work per individual. Regulation of neurogenesis has a strong environmental component, where variability between individuals depends on the experience and life history. Even within a homogenous genetic background and within one experiment, one sex, and standardised laboratory conditions, coefficient of variation for net neurogenesis in rodents is above 0.2, which is reflected in modest heritability of this trait. We have also recorded variation between experiments. Therefore, to achieve reasonable strain mean estimates of neurogenesis, we needed to sample several individuals per strain from at least 3 independent litters born at different times to avoid confusion between potential systemic environmental and genetic influences on the phenotypes.

Inclusion of both sexes in the model, though statistically possible, would require doubling of the animal numbers and become logistically very difficult to conduct. The choice of one sex only had been a compromise dictated by a desire to minimise any additional sources of variation within an operational feasibility of the experiment at the expense of obtaining less comprehensive biological information. The nature of RI strains and our strategy for data collection allow for integration of new information in the future, including measures of neurogenesis in female rats. We are trying to develop automated tissue processing and cell counting, which would enable more efficient quantification of adult neurogenesis in large cohorts of animals. We have modified the last section of the Discussion to be more transparent about our decision:

*Line 693*

“In our study we used only male rats and thus we do not know whether any of the identified associations interacts with sex. Adult neurogenesis has a strong environmental component, where variability between individuals depends on the experience and life history. To avoid interference of systemic environmental errors with genetic sources of variation and obtain reliable estimates of neurogenesis in the HXB/BXH family, we sampled several individuals per strain from at least 3 independent litters born at different time points throughout the data collection period. Inclusion of both sexes may have increased within-strain variation, therefore decreasing power to detect genetic associations. To use sex as a covariate would require a larger number of experimental animals, which was not logistically feasible. Under these circumstances, we chose to use males for compatibility with existing data, as the vast majority of published HXB/BXH phenotypes were measured in young male rats to avoid inter-individual variation due to oestrous cycle. The inbred status of the HXB/BXH family enables integration of additional data at later time points.”

• Line 254: “…presumably generating a non-functional protein”. That is a big presumption. Is there a decrease in TTI2 protein in the mutants? Is there a decrease (or potentially increase?) in TTI2 protein in BN vs. SHR?

Most of commercial antibodies against Tti2 were generated against the human protein, which shares only 71 % identity to rat protein. The antibodies we tested did not recognise rat Tti2 protein, so we could not measure the protein levels within the HXB/BXH panel, progenitor strains or knock-out rats. However, we did not mention in the manuscript that the deletion not only led to a frameshift mutation, but also generated a series of premature stop codons starting at 113^th^ aa position. Full-length Tti2 has 502 amino acids. In addition to the mutational screens performed in yeast, which indicated that cells with truncated Tti2 are not viable, except when truncation is located in the very C-terminus of Tti2, recent reports by Kim et al. (J. Mol. Biol. 2022) and Pal et al. (Cell Rep. 2021), who established structure of TTT and its interactions with R2TP complex using cryo electron microscopy, show that a large portion of the N-terminal domain of Tti2 is interacting with its partners. We replaced the statement about our presumptions in Results as follows:

*Line 293*

“The deletion resulted in a frameshift mutation Glu82Gly, which generated 9 premature stop codons within the N-terminal portion of the protein, truncating Tti2 from 502 to 112 amino acids.”

We also expanded the Discussion:

*Line 521*

“In our present study, we generated a heterozygous SHR-*Tti2*^+/-^ line carrying a frame-shift mutation, which resulted in a series of premature stop codons in the N-terminal domain of *Tti2.* Cryo electron microscopy structure of the human R2TP-TTT complex has revealed that α‑helical regions in the N-terminal domain of Tti2 form an interface for interaction with Tti1 and RUVBL, and are essential for the formation and function of TTT [61,62]. Accordingly, mutational screens in yeast produced viable cells only when truncations were located at the very end of the C-terminus of *Tti2* [71]. Thus we predicted that this modification results in a non-functional protein. *Tti2* mRNA levels in the heterozygous SHR-*Tti2*^+/-^ rats were reduced by 50% compared to wild type littermates, as established by RNA sequencing, implicating nonsense-mediated decay of the mutated mRNA.”

MINOR

• Het-het breeding and Littermate controls for the KO study. 50% knockdown more closely mimics the likely human condition. Why, however, were homozygous mutants not tested? Were they not viable?

All components of TTT complex are essential proteins in yeast. Surprisingly, human patients with loss-of-function mutations in *Tti2* are alive but have severe neurodevelopmental, intellectual and skeletal abnormalities. We speculated that homozygous knockout rats would be viable, but also strongly affected. We did not cross-bred heterozygous animals to obtain homozygotes for two reasons. First, our goal was not to study the absence Tti2 function as such, which would result in major perturbations of many crucial cellular processes, but to mimic an effect of an e-QTL. We believe that a heterozygous model is much more relevant to biology and evolutionary genetics than a full knockout. Current consensus from GWAS is that the majority of genetic variation influencing quantitative traits, including susceptibilities to diseases, act at the level of gene expression, so manipulation of gene dosage is also more applicable to human populations.

The second reason was mainly logistic and statistical. Inclusion of homozygotes in the analyses would have required much more animals for breeding to obtain enough homozygous and wild type animals, which was difficult to achieve without splitting the experiment into multiple batches thus potentially increasing variation. Furthermore, more complex statistical models would have been necessary (including a 2-way mixed-effect model with up to two random effects). This may have impeded detection of subtle phenotypic changes, which we expected. Together, the hypothesis we wanted to test, and 3R animal welfare policies did not justify generation of homozygotes.

• QTL interval should be provided in the abstract, if possible.

We included the QTL interval in the abstract.

• Power analysis for QTL detection of various effect sizes with 30 strains could be provided.

We have provided a graphical summary of power analysis for effect sizes between 0.1 and 1 for heritability in a range between 0.2 and 0.7 in the supplementary Figure S5.

• Line 228: “expression of only one gene…” – do they mean one gene within the chr.16 QTL interval? Or only one gene in the entire genome?

Out of the entire transcriptome data, only *Tti2* expression correlated to both phenotypes in all data sets. There were 46 genes, whose expression correlated to neurogenesis in the hippocampus and simultaneously to serum glucose in any of the peripheral tissues. Out of those, only *Tti2* localised to the QTL region. We have expanded the text in the Results to describe correlations in more detail:

*Line 244*

“We identified 2893 genes whose expression correlated to net neurogenesis in the hippocampus or to serum glucose in at least one peripheral tissue with an absolute value of Pearson’s *r* equal to or larger than 0.4. Among these genes, 3 were located within the common neurogenesis-glucose QTL interval on chromosome 16: Telo2-interacting protein 2 (*Tti2*), Neuregulin 1 (*Nrg1*), and *Mak16*. Serum glucose correlated to expression of *Nrg1* in the muscle (*r* = 0.47), and fat (*r* = 0.44), and to *Mak16* in the muscle (*r* = -0.59), aorta (-0.56), and adrenal gland (*r* = -0.53). *Tti2* was the only gene from the entire transcriptome whose expression correlated significantly to both traits across all data sets (Fig. 2F). Finally, *Tti2* mRNA expression was linked to a multi-tissue cis-eQTL mapping within the neurogenesis-glucose QTL (Fig. 2E). Other genes that had cis-eQTL within the joint QTL interval were *Mak16* in the muscle (LOD = 6.11), and *Saraf* in the kidney (LOD = 4.4) (Table S2). The *Saraf* gene itself is located outside of the phenotype QTL, and its expression did not correlate to physiological phenotypes.”

• Line 698: PMID listed. needs to have the reference inserted.

This omission was corrected.

• Figure legends could be located closer to the figures.

We have now included the figures within the text in the mark-up version of the manuscript.

Reviewer #2

SUMMARY

This is an interesting and thorough manuscript that uses rat RI strains to map a trait locus for adult neurogenesis and identify a strong candidate gene by testing a rat knockout strain generated for this purpose. The primary genetic association is not the strongest, and this particular RI panel has only modest detection power and resolution, but the authors brought sophisticated conditional and expression QTL mapping into the mix to obtain convincing evidence that a candidate gene – Tti2 - is the likely culprit, and then introduced several levels of characterization to examine the relationship between neurogenesis and serum glucose.

• The “proof” that Tti2 gene is the causal gene is almost, but not quite ironclad – more ideal would have been to knockin the most likely (or multiple) missense variants rather than to use the knockout, because of the possibility that loss-of-function is not the sole molecular consequence of the strain variation in Tti2. I do understand, on balance, since there is a clear expression difference in Tti2 between the progenitor strains, that using the ko to test it is reasonable. However, if the authors have or would make compound heterozygotes between the knockout allele and representative RI lines for each progenitor allele (an approach previously referred to as RIST, recombinant inbred segregation testing) this would further strengthen their conclusions and the manuscript.

Given: i) pan-tissue eQTL of *Tti2*; ii) strong correlations between both phenotypes and *Tti2* gene expression in multiple tissues; and iii) evaluation of amino-acid substitutions in *Tti2* as benign by prediction algorithms Polyphen and SIFT, we hypothesised that the expression level of *Tti2* is the most likely culprit of the QTL. The 4.2 Mb QTL interval covers thousands of variants, any of which, singly or in combination, could influence expression of *Tti2* and adjacent genes, particularly *Mak16*, which overlaps *Tti2* locus. We decided therefore that manipulating *Tti2* gene dosage through targeted knock-out would be the optimal strategy to evaluate its role in neurogenesis and metabolism. This approach eliminated possible linkage effects, and allowed quantifying neurogenesis, multiple metabolic phenotypes, and gene expression profiles within stable genotypes. The damaging effect of missense variants—given available data—originally seemed to be less likely. The effect of these variants on Tti2 protein function could probably be evaluated *in vitro*. However, Tti2 is not very strongly conserved protein and the amino acid substitutions in the HXB/BXH panel may not be relevant to genetics of other species.

In our study we were motivated to find out the extent of genetic correlations between neurogenesis and metabolic phenotypes, and possibly identify genes that influence these correlating phenotypes, rather than pinpoint particular causal variation. We agree that our data leaves open questions regarding the QTL itself and the exact mechanism by which *Tti2* influences adult hippocampal neurogenesis and glucose metabolism. Nonetheless, we think that we produced enough evidence to support the conclusion that any variation in *Tti2* that significantly alters its function or expression is likely to have an effect on these phenotypes.

• One minor point – the authors adoption of the p < 0.63 guideline as “suggestive” bothers me. Although I realize it has historical context from earlier QTL rigor standard-seeking guidelines, outside of the QTL world I do not think anyone in their right mind would think that this is suggestive or trending.  Depicting p<0.1 and/or p<0.25 levels seem more realistic ways to report less-than-statistically-significant effects.

Although the *p* = 0.63 threshold is very permissive, it is statistically justified. This definition of suggestive threshold traces back to Eric Lander and Leonid Kruglyak (Genetic dissection of complex traits: Guidelines for interpreting and reporting linkage results. Nat Genet. 1995; 11: 241–247. doi:10.1038/ng1195-241). It has since been a standard widely accepted by the statistical geneticists. The genome-wide corrected *p* value is computed by evaluating the distribution of highest LRS scores generated by a set of 10000 or more random permutations of strain means. Thus, the genome-wide suggestive *p* = 0.63 threshold corresponds, on average, to one or more false QTL discoveries per scan. That is, roughly one-third of scans at this threshold will yield no false positive, one-third will yield one false positive, and one-third will yield two or more false positives. This definition is useful because it calls attention to loci that may be worth following-up. Regions of the genome in which the LRS exceeds the suggestive threshold are particularly useful in combined multicross meta-analyses of traits or composite mapping of multiple traits. If two crosses pick up the same suggestive locus, then that locus may be significant when the joint probability is computed. We would insist on adhering to the convention accepted in the field, because making an exception undermines established practice and therefore might create potentially harmful precedence for the community. Furthermore, the adoption of more stringent suggestive threshold does not influence the conclusions of our study.

Additional changes introduced into the manuscript.

In the revised version of the discussion we added references to several studies published since our first submission. We have also added a supplementary File S1 containing the individual data points for phenotypes measured in SHR-*Tti2*^+/-^ rats and SHR littermates.
